# Assessing effect of best management practices in unmonitored watersheds using the coupled SWAT-BiLSTM approach

**DOI:** 10.1038/s41598-023-44531-7

**Published:** 2023-10-11

**Authors:** Xianqi Zhang, Yu Qi, Haiyang Li, Shifeng Sun, Qiuwen Yin

**Affiliations:** 1 Water Conservancy College, North China University of Water Resources and Electric Power, Zhengzhou, 450046 China; 2 Collaborative Innovation Center of Water Resources Efficient Utilization and Protection Engineering, Zhengzhou, 450046 China; 3 Technology Research Center of Water Conservancy and Marine Traffic Engineering, Zhengzhou, 450046 Henan China

**Keywords:** Hydrology, Pollution remediation

## Abstract

In order to enhance the simulation of BMPs (Best Management Practices) reduction effects in unmonitored watersheds, in this study, we combined the physically-based hydrological model Soil & Water Assessment Tool (SWAT) and the data-driven model Bi-directional Long Short-Term Memory (Bi-LSTM), using the very-high-resolution (VHR) Land Use and Land Cover (LULC) dataset SinoLC-1 as data input, to evaluate the feasibility of constructing a water environment model for the Ba-River Basin (BRB) in central China and improving streamflow prediction performance. In the SWAT-BiLSTM model, we calibrated the top five SWAT parameters sorted by P-Value, allowing SWAT to act as a transfer function to convert meteorological data into base flow and storm flow, serving as the data input for the Bi-LSTM model. This optimization improved the Bi-LSTM's learning process for the relationship between the target and explanatory variables. The daily streamflow prediction results showed that the hybrid model had 9 regions rated as "Very good," 2 as "Good," 2 as "Satisfactory," and 1 as "Unsatisfactory" among the 14 regions. The model achieved an NSE of 0.86, R^2^ of 0.85, and PBIAS of −2.71% for the overall daily streamflow prediction performance during the verification period of the BRB. This indicates that the hybrid model has high predictive accuracy and no significant systematic bias, providing a sound hydrodynamic environment for water quality simulation. The simulation results of different BMPs scenarios showed that in the scenarios with only one BMP measure, stubble mulch had the best reduction effect, with average reductions of 17.83% for TN and 36.17% for TP. In the scenarios with a combination of multiple BMP measures, the combination of stubble mulch, soil testing and formula fertilization, and vegetative filter strip performed the best, achieving average reductions of 42.71% for TN and 50.40% for TP. The hybrid model provides a novel approach to simulate BMPs' reduction effects in regions without measured hydrological data and has the potential for wide application in BMP-related decision-making.

## Introduction

With the development of water environment management technology and practices, it is widely recognized that besides direct discharge of wastewater, the main causes of water quality deterioration and eutrophication in rivers and lakes are due to human activities, including agricultural activities and urban emissions, which disrupt the structure and functioning of watershed ecosystems and degrade the intrinsic elements, leading to non-point source pollution, such as carbon, nitrogen, and phosphorus^1^. In some data-scarce regions, modeling the water environment to assess the effectiveness of BMPs is a challenging task^2^. Due to limited hydrological monitoring stations and the increasing and diversified demand for hydrological data with socio-economic development, the issue of data scarcity is expected to persist. In this context, the development of improved watershed hydrological models to enhance the simulation of streamflow and water quality in data-scarce areas has become an urgent necessity. NPS pollution is characterized by a wide range of sources, strong randomness, and high concentrations of pollutants, making its control strategies significantly different from point source pollution. Currently, BMPs have been proven to be one of the most effective measures for managing NPS pollution^3^. BMPs are divided into agricultural BMPs and structural BMPs^4^, which manage the water environment through engineering and management measures, respectively. In order to achieve environmental goals for non-point source pollution control, such as reducing nitrogen and phosphorus loads by 30%, a certain amount of economic cost is required for construction and management. However, under the same economic cost, different spatial configuration schemes have different environmental benefits. To develop the most efficient plan (minimizing economic costs and maximizing pollution reduction), it is essential to quantitatively evaluate its benefits before implementation^5^. Therefore, enhancing the understanding of BMPs' performance is crucial.

Watershed models are mathematical representations of hydrological, ecological, erosion, and nutrient cycling processes within a watershed. Based on their approach and the processes they simulate, they are typically classified as empirical models and physical models (or process models)^6^. Process models, also known as hydrological process-based models, are built on hydrological processes such as rainfall, evaporation, infiltration, and runoff, and they describe the transport and transfer of pollutants using water as a carrier. Additionally, they simulate processes like vegetation growth, soil erosion, and nutrient cycling. Compared to empirical models, process models can better describe the migration paths and transformation mechanisms of pollutants^7^. Therefore, in recent years, physical models including Area Nonpoint Source Watershed Environment Simulation (ANSWERS), LOAD ESTimator (LOADEST), SWAT and Hydrological River Basin Environment Assessment Model (HydroBEAM) have been widely used in various water environment studies^8–11^. These models have different structures and mechanisms and use different equations to describe BMPs^12^. In general, most models tend to focus on simulating only one or a few processes within the watershed, such as hydrology, soil erosion, or nutrient cycling, with only a few models, including SWAT, considering various processes within the watershed^13^. Moreover, SWAT's built-in equations provide a more detailed description of agricultural activities and BMPs^14^. Previous research has shown that SWAT can effectively study the performance of BMPs due to changes in hydro-meteorological characteristics, land use and land cover (LULC), and soil properties^10^. However, SWAT requires various types of input data (such as precipitation, temperature, evaporation, topography, soil properties, and LULC), demanding higher temporal and spatial resolutions, and its performance is highly dependent on the quality of input data and parameters^15^. Additionally, the calibration of parameters in SWAT is subject to complex uncertainties due to the intricate issue of equifinality^16^, increasing the modeling difficulty and consuming a significant amount of researchers' time.

In recent years, data-driven models have been widely applied in various water environment studies, and their reliability has been validated^17^. Essentially, data-driven models aim to derive the linear or nonlinear relationships between explanatory and target variables based on a large amount of input data, without considering the physical characteristics of the variables. Bi-LSTM model is one type of data-driven model, consisting of two opposite-directional Long Short-Term Memory (LSTM) models, and its performance has been shown to outperform single-directional LSTM models in many aspects^18,19^. Using Bi-LSTM to simulate watershed runoff can bypass the complex and uncertain calibration process, significantly reducing modeling difficulty^20^. However, its main challenge lies in the high requirement for representativeness of training data,once events fall outside the range of the training data, the predictive performance of the model will deteriorate significantly^21^. Additionally, data-driven models like Bi-LSTM cannot account for the impact of spatiotemporal characteristics of rainfall on the runoff generation process in the watershed. For example, Jiang et al.^22^ observed that when the rainfall center is close to the outlet of the watershed, the water level at the outlet section rises rapidly. This is because such data-driven models use rainfall time series from different meteorological stations as input data, overlooking the potential influence of spatial variability on runoff variations in the study area.

However, whether it is conceptual hydrological models or machine learning models, their performance in data-scarce regions remains unsatisfactory. In a streamflow simulation study of a sub-basin in the Tonle Sap Basin of Cambodia, where no actual measured data were available, researchers calibrated and validated the SWAT model using daily runoff observations at the watershed outlet. After numerous attempts, the model achieved an NSE of 0.38 and a PBIAS of -78.38% for daily streamflow simulation results during the validation period. This indicates that hydrological models based on physical processes, such as SWAT, perform inadequately in data-scarce regions^23^. Moreover, due to the absence of observed hydrological data for training, machine learning models cannot be directly applied to data-scarce watersheds. In this study, to enhance streamflow and water quality simulation in data-scarce watersheds, and to overcome the limitations inherent in both conceptual hydrological models and machine learning models, we developed a coupled SWAT-BiLSTM model. In this hybrid model, the SWAT model serves as a transfer function, combining meteorological information, including temperature, precipitation, wind speed, and humidity, with topographic, soil, and LULC data to transform them into two hydrological variables: baseflow and quickflow. Bi-LSTM, on the other hand, captures linear or nonlinear underlying relationships between the two hydrological variables (explanatory variables) and observed streamflow data (target variable), ultimately enabling streamflow prediction in data-scarce watersheds. This provides a new approach for modeling water environments in areas without measured hydrological data, thus reducing the difficulty in evaluating the reduction effects of BMPs schemes in these regions.

In this study, to assess the reduction effects of different BMPs scenarios in areas without measured hydrological data, we selected the BRB in Shaanxi Province, China, which is known for its severe water pollution, as the case study area. The objectives of this study were as follows: (1) to establish a hybrid model combining SWAT and Bi-LSTM and use it to predict the streamflow in the assumed data-scarce areas; (2) to evaluate the predictive performance of the hybrid model in different regions of BRB; (3) to simulate and evaluate the reduction effects of different BMPs scenarios.

## Materials and methods

### Study area

**Figure 1:**
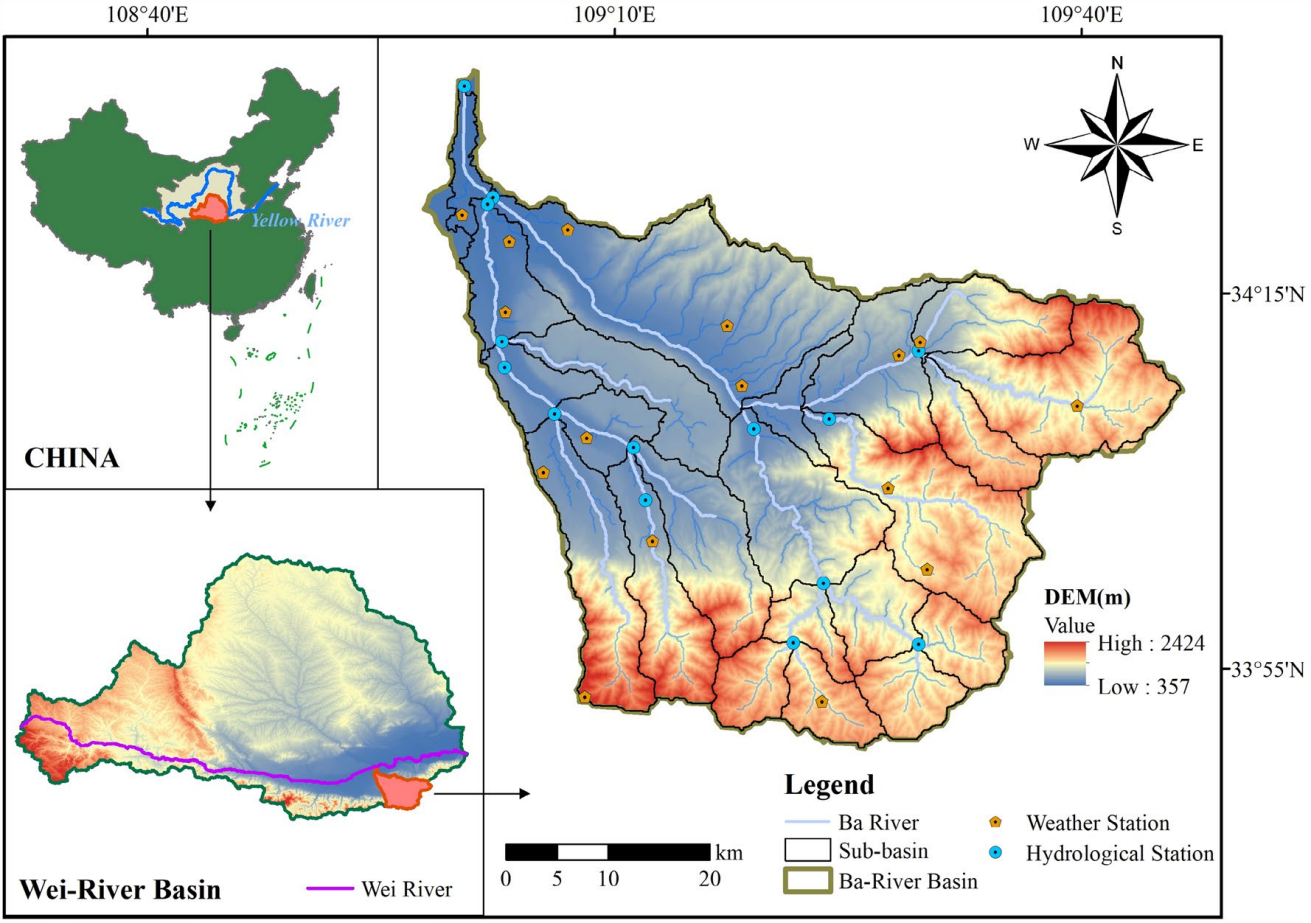
Location and sub-basin delineation of Ba-River Basin. The figure is created using ArcMap 10.2, URL: https://www.arcgis.com.

The Ba-River is located in the southeastern part of Xi'an City, Shaanxi Province, China. It originates from the northern slope of the Qinling Mountains, north of Lantian County, and flows through Baqiao District and Weiyang District before joining the Yellow River's main tributary, the Wei-River, in Gaoling County. The river has a total length of 109 km and is the largest tributary on the south bank of the Wei River. The Ba-River Basin (33°50′ N–34°27′ N, 109°00′ E–109°47′ E) covers an area of 2581 km^2^, with its topography mainly composed of mountains, ranging in elevation from 357 to 2424 m (Fig. 1). The southern and eastern parts of the basin are mainly covered by forests, while the central part is dominated by extensive farmland. Villages are distributed along both sides of the river, and the northern part is primarily used for urban construction. The predominant soil types in the basin are yellow–brown soil and brown soil. The BRB experiences a warm temperate semi-humid continental monsoon climate with significant seasonal characteristics. The majority of heavy rainfall occurs from July to September, often in the form of continuous rainy days and heavy storms, with a spatial distribution that is generally more rain in the south and less in the north. The total annual precipitation ranges from 502 to 873 mm, with an average of 697 mm over multiple years. The average annual temperature in the basin is between 13.0 and 14.8 ℃, with a multi-year average of 13.7 ℃. The average annual evaporation is 776 mm^24^. The overall groundwater quality in the BRB is good, but in the Ba-River Ecological Zone, human activities have led to fluoride and total coliform exceeding the standards in some areas, resulting in poor water quality. The pollution sources in different locations of the Ba-River are influenced by the hydrological characteristics, uneven population distribution, and regional economic disparities. The upper and middle reaches of the river are mainly affected by livestock farming discharge, domestic sewage, and agricultural pollution, while the lower reaches receive concentrated urban domestic sewage and industrial wastewater. In recent years, with the construction of sewage treatment facilities in the BRB, the pollution load from point sources has been continuously reduced, but NPS pollution has become increasingly prominent^25^. Therefore, there is an urgent need to conduct research on the effectiveness of NPS pollution emission control schemes in this area.

### Soil and water assessment tool (SWAT)

**Figure 2:**
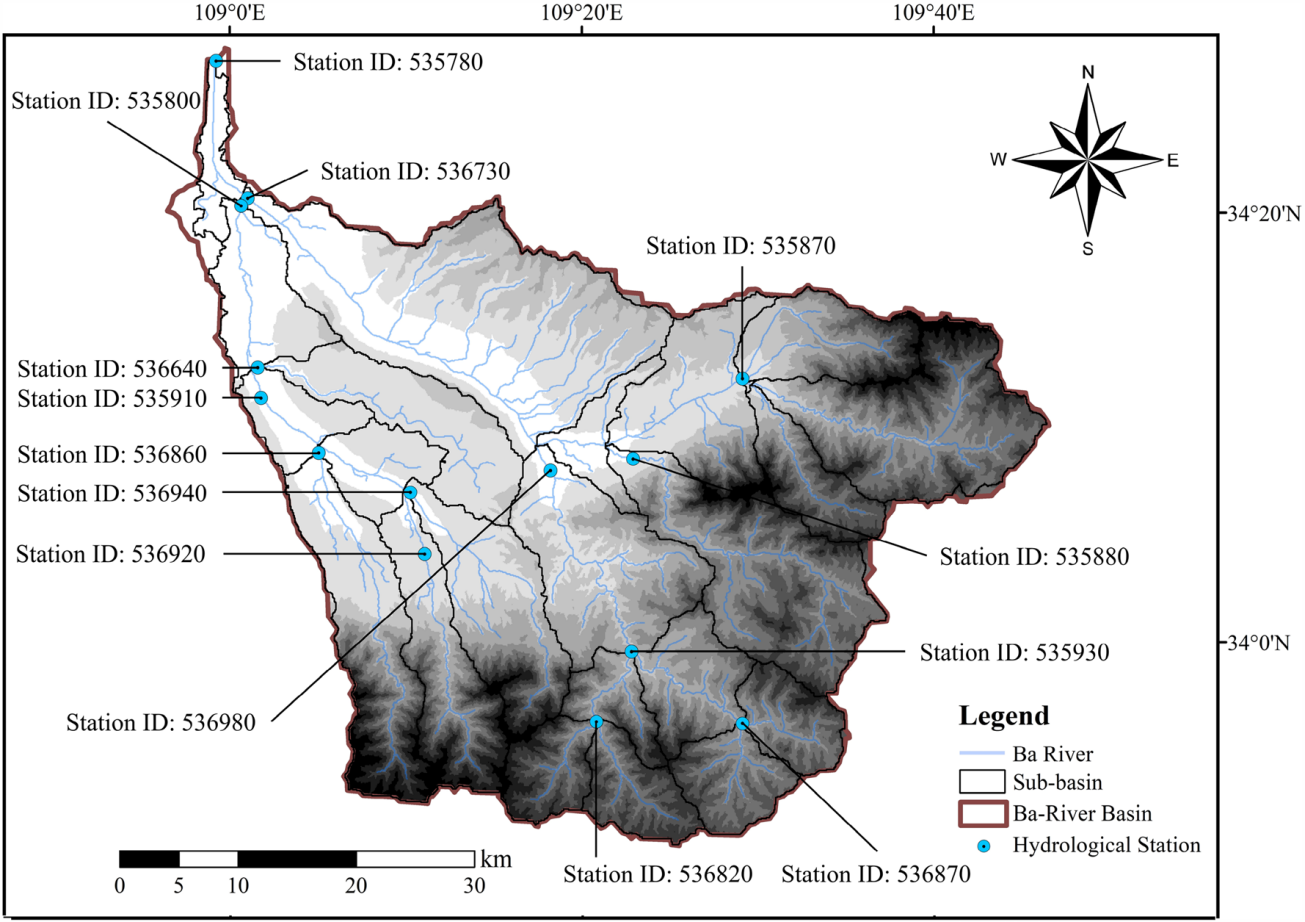
Geographical location of hydrological stations. The figure is created using ArcMap 10.2, URL: https://www.arcgis.com.

SWAT is a physically-based semi-distributed watershed hydrological model developed by Dr. Arnold of the Agricultural Research Service (ARS) of the United States Department of Agriculture (USDA). Initially, SWAT was applied to large-scale and complex watersheds with different soil types, land use, and management conditions to predict and evaluate the long-term impacts of human activities, such as land use management, on the water cycle, sediment, and agricultural pollutant transport in the watershed^26^. SWAT model is based on the Simulator for Water Resources in Rural Basins (SWRRB) model and incorporates several characteristics of ARS models. The improvement of the SWRRB model originated from the daily rainfall hydrological model of Chemicals, Runoff, and Erosion from Agricultural Management Systems (CREAMS). In the late 1980s, for water quality assessment, the SWRRB model incorporated pesticide components from the Groundwater Loading Effects of Agricultural Management Systems (GLEAMS) model, the SCS curve method, and newly developed sediment yield calculation equations to address watershed management issues. The SWAT model can simulate the movement of water in evapotranspiration, groundwater, and soil based on empirical equations and the principle of water balance. It not only simulates the water cycle process but also studies the processes of soil erosion, nutrient transport, pesticide, and pathogen cycling using the water cycle as a carrier. In recent years, the model has also been widely used in various aspects such as non-point source pollution detection and control, mechanistic process exploration simulation, and spatial–temporal distribution of pollution load^27,28^. In the SWAT modeling process, a watershed is first divided into several sub-basins, and then, combining with data such as land use types and soil types, the sub-basins are further divided into different Hydrological Response Units (HRUs). The Soil Conservation Service (SCS) method is used to independently calculate water infiltration and surface runoff in each HRU, and the surface water is calculated at the outlet of the sub-basin, and finally, the routing process is calculated using a simulation computation method. Table 1 shows the data and sources used to establish the SWAT model in the BRB. Figure 2 illustrates the Station ID of the 14 hydrological stations in the SWAT database and the corresponding geographical locations.

**Table 1:** Data inputs for the SWAT model.

Data	Data source
DEM	https://www.gscloud.cn/
LULC dataset	https://doi.org/10.5281/zenodo.7821068/ (SinoLC-1)
Soil maps	Institute of Soil Science, Chinese Academy of Sciences
River networks	National Geomatics Center of China (NGCC)
Daily rainfall records (Jan 2015–Dec 2022) (15 stations)	National Oceanic and Atmospheric Administration (NOAA) https://www.noaa.gov/
Daily temperature records (Jan 2015–Dec 2022) (15 stations)	
Daily streamflow records (Jan 2015–Dec 2022) (14 stations)	Ministry of Water Resources of the People's Republic of China

**Figure 3:**
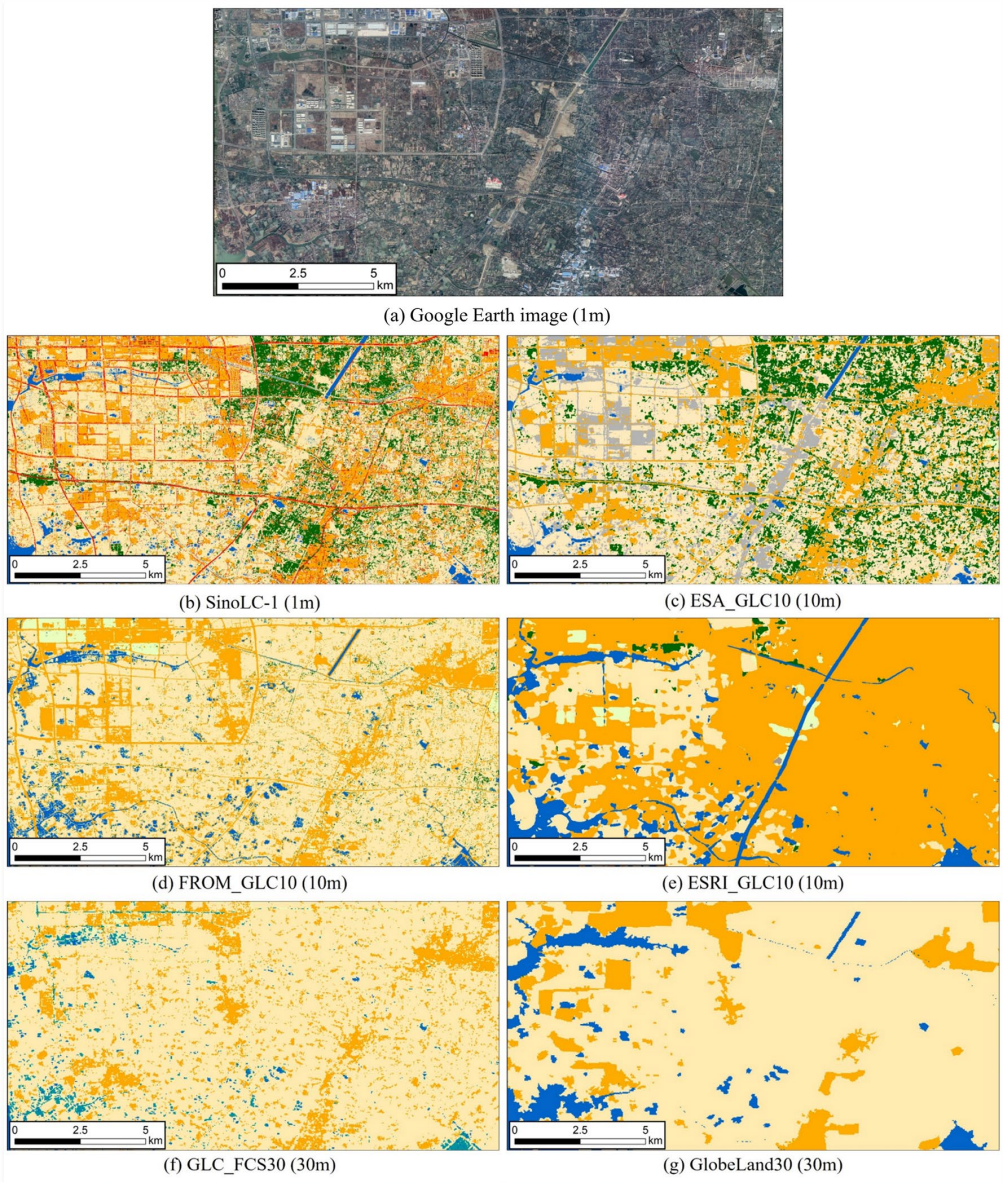
Demonstration of the visual comparison for Changzhou City, Jiangsu Province. The VHR remote sensing image in the figure is from Google Earth 2021.

It is worth mentioning that this study used the first 1-m resolution national-scale land-cover map of China created with the deep learning framework to improve modeling accuracy. This dataset is derived from the State Key Laboratory of Information Engineering in Surveying, Mapping, and Remote Sensing (LIESMARS), Wuhan University, with a resolution of 1 m^27,28^. Figure 3 shows the comparison of SinoLC-1 with other LULC datasets at a larger spatial scale. Based on the analysis of the VHR satellite image in Fig. 3a, the land cover performance of ESRI_GLC10 in Fig. 3e and GlobeLand30 in Fig. 3g is the most blurred, with farmland, buildings, and forests in urban areas being severely confused. GLC_FCS30 performs the worst in terms of forest cover, transportation roads, rivers, and runoff. FROM_GLC10 shows accurate performance on water bodies (such as artificial lakes and rivers), but its performance in forest cover types does not meet expectations. ESA_GLC10 relatively performs better compared to other comparative products, but its performance in water bodies is still inadequate. In comparison, SinoLC-1 has the best overall performance, accurately representing fine details of land cover such as small rivers, artificial lakes, small ponds, vegetation, and buildings. It can precisely identify the boundaries of different land use types, which significantly reduces the phenomenon of confusion between different LULC types during the SWAT modeling process and contributes to the accurate delineation of HRUs. To quantitatively assess the performance differences between SinoLC-1 and five other widely used large-scale land cover products, a total of 106,852 random samples extracted from each LULC product were compared and analyzed against official land survey reports provided by the Chinese government. The validation results indicated that SinoLC-1 achieved an overall accuracy of 91.7% and a kappa coefficient of 0.7595, indicating a high level of consistency between SinoLC-1 and actual LULC data. Information for the other five LULC products is presented in Table 2^28^.

**Table 2:** Information for the comparative land-cover products.

Name	Resolution (m)	Version and Timeline	Number of LULC type	Overall accuracy (%)
ESA_GLC10	10	V2020	11	73
FROM_GLC10	10	V2017	10	74
ESRI_GLC10	10	V2020	10	85
GLC_FCS30	30	V2020	16	83
GlobeLand30	30	V2020	10	86

To simulate the effects of BMPs, the BRB was divided into 23 sub-basins based on the terrain and real river network vector data, using a threshold of 50 km^2^. Based on the SinoLC-1 dataset and soil type data, 21 sub-basins were further subdivided into 713 HRUs by setting thresholds for LULC, soil type, and slope at 13%, 20%, and 20%, respectively. The daily runoff records from the hydrological station at the BRB outlet were used to calibrate and validate the SWAT model. The calibration period was from January 1, 2015, to December 31, 2019, and the validation period was from January 1, 2020, to December 31, 2022. Considering the issue of equifinality involved in the model calibration process, simultaneous calibration of a large number of parameters can lead to significant uncertainty^16^. Based on previous research in the WRB region^29^, we selected different types of parameters ranked in the top five P-Values in sensitivity analysis for calibration. The calibrated parameters and their values are presented in Table 3. This process was performed using the Sequential Uncertainty Fitting 2 (SUFI-2) algorithm built into SWAT-CUP.

**Table 3:** Calibrated parameters in SWAT.

Type	Sensitivity ranking	Parameter	Definition	P-value	Calibrated value
Runoff	1	ALPHA_BF	Baseflow α coefficient	0.016	0.045
	2	ESCO	Soil evaporation compensation factor	0.017	0.78
	3	GWQMN	Shallow groundwater runoff coefficient	0.028	1195
	4	GW_DELY	Groundwater lag factor	0.035	24.02
	5	SOL_Z	Soil depth	0.044	2560
TN	1	CN2	SCS runoff curve coefficient	0.066	87
	2	NPERCO	nitrogen permeability coefficient	0.083	0.245
	3	USLE_P	Soil and water conservation measures factor	0.099	0.43
	4	CDN	Denitrification index rate factor	0.183	1.12
	5	SDNCO	Soil water content thresholds for denitrification	0.271	0.805
TP	1	USLE_P	Soil and water conservation measures factor	0.060	0.43
	2	RCHRG_DP	Deep aquifer permeability coefficient	0.064	0.046
	3	CN2	SCS runoff curve coefficient	0.070	87
	4	PHOSKD	Soil phosphorus partition coefficient	0.097	156.62
	5	PPERCO	Phosphorus permeability coefficient	0.108	10.00

### Bi-LSTM

**Figure 4:**
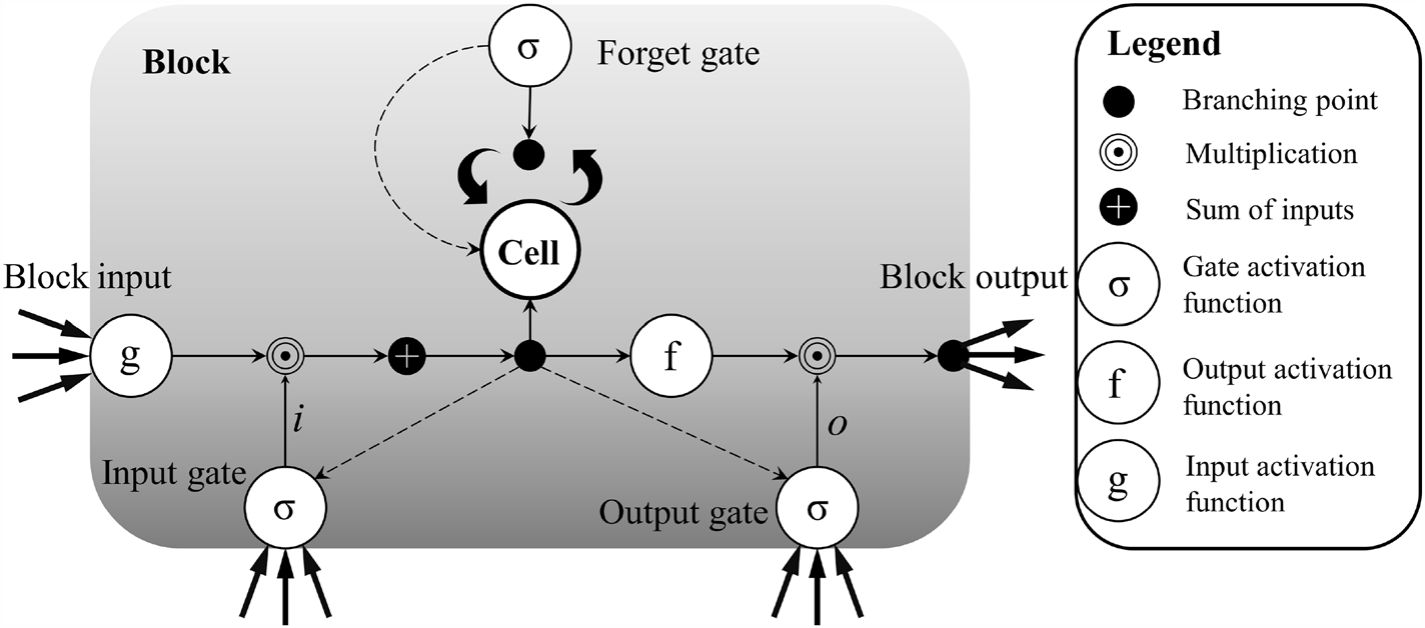
Illustration of an LSTM block.

LSTM is an improved type of Recurrent Neural Network (RNN) that addresses the issue of long-term dependencies encountered in traditional RNNs^30^. In the structure of LSTM, the hidden layer neurons are equipped with input gates, forget gates, and output gates. These gates, determined by Sigmoid functions and element-wise multiplication, decide which information should be remembered, giving LSTM the ability of long-term memory and effectively overcoming the vanishing and exploding gradient problems encountered in traditional RNNs^31^. The internal mechanism of a single LSTM neuron is illustrated in Fig. 4, and the mechanisms of the three gates are represented by the following equations:

Forget gate:

1 $${f}_{t}=\sigma \left({W}_{f}\cdot \left[{h}_{t-1},{x}_{t}\right]+{b}_{f}\right)$$

Input gate:

2 $${i}_{t}=\sigma \left({W}_{i}\cdot \left[{h}_{t-1},{x}_{t}\right]+{b}_{i}\right)$$

3 $${\widetilde{C}}_{t}=\mathit{tanh}\left({W}_{C}\cdot \left[{h}_{t-1},{x}_{t}\right]+{b}_{C}\right)$$

4 $${C}_{t}={f}_{t}\odot {C}_{t-1}+{i}_{t}\odot {\widetilde{C}}_{t}$$

Output gate:

5 $${o}_{t}=\sigma \left({W}_{O}\cdot \left[{h}_{t-1},{x}_{t}\right]+{b}_{O}\right)$$

6 $${h}_{t}={O}_{t}\odot \mathit{tanh}\left({C}_{t}\right)$$

Here, ${f}_{t}$, ${i}_{t}$, and ${o}_{t}$ represent the forget gate, input gate, and output gate, respectively; ${C}_{t}$ is the memory cell; ${h}_{t}$ is the output of the neuron's short-term memory at time $t$; ${\widetilde{C}}_{t}$ represents the memory from the new input; $h$ is the hidden vector; $\sigma$ is the activation function; $W$ is the weight matrix; $b$ is the bias term; [M, N] denotes the concatenation of two vectors; ⨀ represents element-wise multiplication.

Although LSTM has the ability of long-term memory, it can only perform forward learning and extract information from unidirectional time series, which limits its learning capacity. Bi-LSTM, by stacking two LSTM networks in opposite directions, utilizes time series data twice and can more fully explore the potential correlation information between the input variables and the target variables. To determine the appropriate network structure and hyperparameters for optimizing Bi-LSTM's performance, we used the Firefly optimizer (FHO) to find the best combination of different hyperparameters. The population size, maximum number of iterations, extinction coefficient, and attraction coefficient were set to 60, 1000, 0.7, and 4, respectively. FHO is an evolutionary algorithm inspired by the foraging behavior of the Black kite, the Maroon Oriole, and the Brown Falcon, with strong global search capabilities^32^. Compared to traditional gradient-based optimization algorithms, FHO does not rely on the gradient information of the objective function, making it suitable for optimizing problems involving non-continuous, non-smooth, and even black-box functions^33^. After conducting multiple experiments by using the hyperparameters of Bi-LSTM as the search dimensions of FHO, the optimal Bi-LSTM model was found to have 512 neurons in the first hidden layer and four dense layers with 256, 78, 32, and 1 neurons, respectively. To prevent overfitting, a Dropout rate of 0.3 was set for the model. Additionally, Rectified Linear Unit (ReLU) was used as the activation function for the hidden layers to reduce computation and avoid the vanishing gradient problem^34^.

### Coupling SWAT with Bi-LSTM

**Figure 5:**
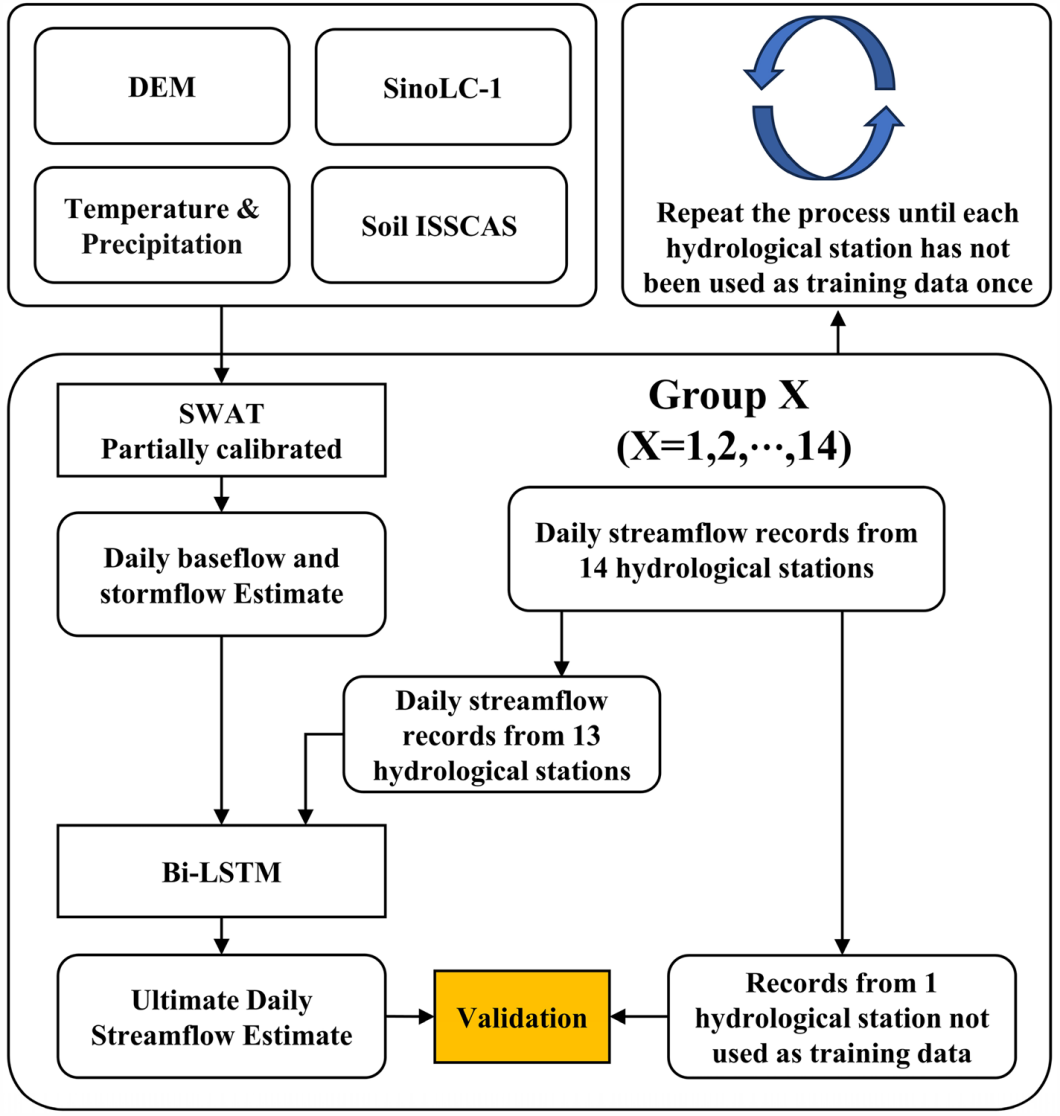
Model training and validation flowchart.

In this study, to establish a water environment model in data-scarce regions, we used a hybrid model combining SWAT and Bi-LSTM. SWAT is responsible for simulating baseflow and stormflow generated by precipitation events. The simulated results are then used to train the Bi-LSTM model to predict daily streamflow during the simulation period. In this process, only some parameters of SWAT are calibrated, which significantly reduces the uncertainty and unnecessary time and effort invested in the modeling process while ensuring model performance. Essentially, SWAT acts as a transfer function, transforming input variables such as terrain, soil type, weather, and LULC into two output variables: baseflow and stormflow. To validate the performance of the hybrid model in different regions, we adopted a cross-validation approach. There are a total of 14 hydrological stations in the BRB. We iteratively excluded one station and used the daily streamflow data from the remaining stations to train an independent Bi-LSTM model. Finally, the excluded station was used to verify the performance of the trained model, and the performance of each model in the target region was obtained. In this process, a total of 14 Bi-LSTM models were trained. The flowchart of this approach is shown in Fig. 5.

### Model performance evaluation

Three metrics were used to evaluate the performance of the established SWAT and Bi-LSTM hybrid model in predicting streamflow in data-scarce regions. They are the Nash–Sutcliffe efficiency (NSE), coefficient of determination (R^2^), and Percent Bias (PBIAS). NSE and R^2^ reflect the degree of collinearity between observed and simulated values, while PBIAS reflects the systematic bias between simulated and observed values. Their calculation formulas are as follows:

7 $$NSE=1-\frac{{\sum }_{i=1}^{N}{\left({M}_{i}-{S}_{i}\right)}^{2}}{{\Sigma }_{i=1}^{N}{\left({M}_{i}-{\overline{M} }_{i}\right)}^{2}}$$

8 $${R}^{2}=1-\frac{{\Sigma }_{i=1}^{N}{\left({M}_{i}-{S}_{i}\right)}^{2}}{{\Sigma }_{i=1}^{N}{\left({M}_{i}-{\overline{M} }_{i}\right)}^{2}{\Sigma }_{i=1}^{N}{\left({S}_{i}-{\overline{S} }_{i}\right)}^{2}}$$

9 $$PBIAS=\frac{\sum_{i=1}^{n}100\left({M}_{i}-{S}_{i}\right)}{\sum_{i=1}^{n}{M}_{i}}$$

where, ${M}_{i}$ and ${S}_{i}$ represent the observed values and simulated values, respectively; ${\overline{M} }_{i}$ and ${\overline{S} }_{i}$ represent the mean of observed values and the mean of simulated values, respectively. Furthermore, to demonstrate the daily streamflow prediction performance of the hybrid model trained based on neighboring areas when different regions in the BRB became assumed no-data regions, we utilized the ranking method presented in Table 4 to assess the model performance in different areas. This ranking criteria is derived from previous research that employed machine learning models for simulating and predicting water environments^35^. However, in the SWAT-BiLSTM coupled model, SWAT is used with default parameters solely as a transfer function. Therefore, we did not opt for performance grading standards biased towards conceptual hydrological models. During the evaluation process, model performance is ranked based on the worse of the two metrics.

**Table 4:** Performance ranking criteria.

Level	NSE	PBIAS (%)
Very good	≥ 0.70	≤ 25
Good	0.50 ≤ value < 0.70	≤ 50
Satisfactory	0.30 ≤ value < 0.50	≤ 70
Unsatisfactory	< 0.30	> 70

### BMP scenario settings

BMPs have been widely used for the prevention and control of NPS pollution and have shown significant effects. However, different BMPs have distinct spatial variations in their reduction effects on NPS pollution, requiring tailored management measures that suit the actual characteristics of the watershed. Based on the natural characteristics of the BRB (LULC, soil types, slope, and topography), socio-economic development (population density and water quality), and current NPS pollution status, we set two major categories of measures: Agricultural BMPs and Structural BMPs. Agricultural BMPs include formula fertilization by soil testing and stubble mulch, while Structural BMPs encompass vegetative filter strips and grassed waterways. The pollutant reduction effects of these four BMPs have been proven in previous studies^36^. Table 5 presents the information on each BMP and the parameters that need adjustment in SWAT.

**Table 5:** The description and the simulation method of each BMP.

BMPs code	BMP type	Category	Simulation method and parameters
B1	Formula fertilization by soil testing	Agricultural BMPs	FRT**_**KG
B2	Stubble mulch		Harvest only, CN2, USLE_P, USLE_C and OV_N
B3	Vegetative filter strip	Structural BMPs	VFS routine or FILTERW
B4	Grassed waterway		GWATD, GWATW, GWATSPCON, GWATN, The channel erodibility factor (CH_COV1) and channel cover factor (CH_COV2)

We obtained relevant information on fertilizer application in the watershed through on-site field surveys of farmers. The cultivated area in the BRB is 401.01 km^2^, with main crops being wheat and corn. The commonly used fertilizers are nitrogen-based (mainly urea and ammonium bicarbonate) and phosphate-based (mainly calcium superphosphate). The average application rates of chemical fertilizers for wheat and corn are 1125 kg/ha and 750 kg/ha, respectively. The total annual application of chemical fertilizers in the cultivated land of the watershed is 50,126 tons. The fertilization method is mainly broadcasting, resulting in lower fertilizer utilization efficiency, and significant nitrogen and phosphorus nutrient loss due to rainfall runoff. To address this, we implemented the measure of formula fertilization by soil testing to reduce the amount of chemical fertilizers while maintaining crop yields and reducing pollution loads^37^. Formula fertilization by soil testing was achieved by reducing FRT_KG by 20% in SWAT parameters. Stubble mulch is an effective agricultural measure in reducing nitrogen and phosphorus losses. The pollutant reduction mechanisms of stubble mulch primarily come from two processes: (1) stubble mulch favors the accumulation of organic matter in the soil, improving soil water-holding capacity, reducing soil erosion, and lowering the risk of nitrogen and phosphorus nutrient loss,(2) stubble mulch reduces soil permeability and promotes the accumulation of reactive substances, effectively facilitating denitrification processes in the soil, leading to more nitrogen being released in the form of gas rather than being discharged into the rivers^38^. Stubble mulch was implemented by adjusting SWAT parameters as follows: USLE_P was set to 0.29, USLE_C was set to 0.7, and OV_N was set to 0.3. Vegetative filter strip (VFS) refers to vegetated areas with gentle slopes that slow down surface runoff and remove pollutants and sediments from runoff through vegetation interception and soil infiltration^39^. In this study, VFSRATIO was set to 40, VFSCON was set to 0.5, and VFSCH was set to 0. Grassed waterways mainly use vegetation to trap and store runoff, reduce flow velocity, and control the migration and transformation of pollutants in runoff, thereby reducing pollutant levels^40^. The width of the grassed waterways was set to 5 m. VFS and grassed waterways were implemented along the entire length of the river reach. In this study, we conducted individual scenario simulations and combination scenario simulations for the four BMPs (Table 6). In all combination scenario simulations, each BMP was set as in the individual scenario simulations. In this study, the pollutant reduction effect of BMPs was expressed as the annual removal rate, defined as follows:

**Table 6:** Scenario settings.

S1	S2	S3	S4	S5	S6	S7	S8	S9
B1	B2	B3	B4	B1 & B2	B1 & B3	B1 & B4	B1, B2 & B4	B1, B2 & B3

10 $$r=\frac{LOA{D}_{Pre}-LOA{D}_{post}}{LOA{D}_{Pre}}\times 100\%$$

Here, $LOA{D}_{Pre}$ represents the annual pollution load before implementing the BMP, and $LOA{D}_{post}$ represents the annual pollution load after implementing the BMP.

## Results and discussion

### Simulation performance comparison

**Figure 6:**
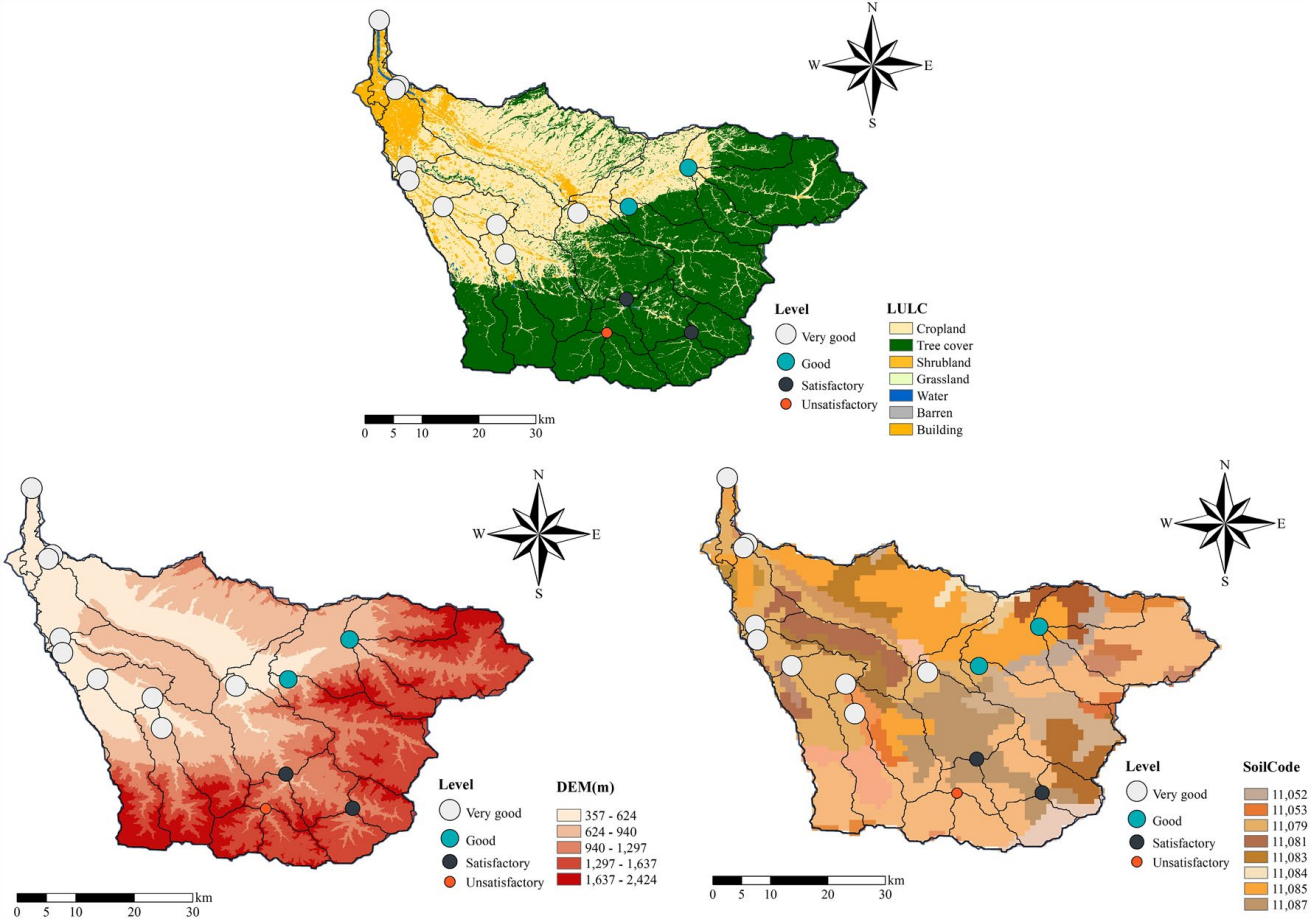
Hybrid model performance rankings in 14 regions.

In this study, we sequentially exclude the data of one hydrological station from the training dataset and use it to validate the model's streamflow prediction performance in a hypothetical area with no measured data. This process generated 14 groups of training dataset, each containing data from 13 hydrological stations, along with corresponding validation data. Table 7 compares the performance of the hybrid model in predicting daily streamflow in different regions of the BRB during the calibration period (January 1, 2015, to December 31, 2017) and the validation period (January 1, 2018, to December 31, 2022). In the validation data from these 14 stations, the absolute values of PBIAS for more than half of the stations are below 10%, with only three stations exceeding 15%. This indicates that the hybrid model's predictions of streamflow in areas without data did not exhibit significant systematic biases. However, there are still four stations with absolute PBIAS values exceeding 10%, and two of them even exceed 20%, suggesting that the hybrid model's predictive performance of daily streamflow is relatively poor in certain specific areas due to spatial factors such as terrain, LULC, and soil types. The performance ratings of the hybrid model in regions with different soil types, terrains, and LULC are shown in Fig. 6. The soil names and brief descriptions corresponding to the soil codes are displayed in Table 8.

**Table 7:** Comparison of daily streamflow prediction performance among the 14 hydrological stations.

Station ID	Coordinates	Calibration period (2015–2019)			Validation period (2020–2022)		
		*NSE*	*R*^2^	*PBIAS* (%)	*NSE*	*R*^2^	*PBIAS* (%)
535910	34°10′26″ N, 109°3′22″ E	0.88	0.89	−2.54	0.86	0.85	−2.71
535800	34°19′7″ N, 109°2′4″ E	0.81	0.84	4.61	0.69	0.76	4.68
535870	34°11′41″ N, 109°29′51″ E	0.64	0.69	12.94	0.53	0.60	13.27
535780	34°25′39″ N, 109°0′32″ E	0.84	0.86	−9.56	0.81	0.84	−10.41
535880	34°7′58″ N, 109°23′54″ E	0.67	0.72	7.13	0.62	0.63	12.98
535930	33°59′14″ N, 109°23′58″ E	0.53	0.52	−39.54	0.49	0.47	−41.72
536640	34°11′48″ N, 109°3′22″ E	0.89	0.91	1.92	0.87	0.88	2.13
536730	34°19′28″ N, 109°2′26″ E	0.86	0.89	3.67	0.81	0.83	4.03
536820	33°56′3″ N, 109°22′6″ E	0.23	0.21	−21.06	0.20	0.19	−22.39
536860	34°8′0″ N, 109°6′36″ E	0.92	0.92	1.34	0.89	0.90	1.91
536940	34°6′17″ N, 109°11′41″ E	0.75	0.78	9.16	0.74	0.71	9.36
536870	33°56′4″ N, 109°30′7″ E	0.44	0.48	18.72	0.42	0.45	17.62
536920	34°3′31″ N, 109°12′31″ E	0.81	0.82	−6.65	0.80	0.79	−6.73
536980	34°7′23″ N, 109°19′22″ E	0.87	0.91	7.03	0.83	0.85	6.98

**Table 8:** Soil type information.

Soil code	Name	Description
11052	Coarse-grained soil	The usual display rapid runoff characteristics
11053	Yellow–brown soil	Poor permeability, difficult cultivation, and high surface runoff
11079	Dark brown soil	Poor permeability, high surface runoff
11081	Black loam	The humus layer is deep, with good tillage suitability, and all of it has been cultivated soil
11083	Brown soil	Good water and nutrient retention, suitable for the growth of various crops, and it is an important arable soil
11084	Gray calcareous soil	The soil is sandy loam, with severe sandification and relatively high permeability
11085	Yellow clay	When facing rainfall, it typically exhibits a relatively uniform infiltration runoff characteristic
11087	Red soil	Good permeability and drainage capability often lead to a reduction in surface runoff

Based on the analysis of terrain, soil type, and LULC data, in the test areas ranked as "Very good," imperviousness ranged from 19 to 54%, and forest cover ranged from 2 to 27%. In the test areas rated as "Good," imperviousness ranged from 18 to 51%, and forest cover ranged from 2 to 35%. Since most of the hydrological data used to train the Bi-LSTM model came from urbanized areas with relatively flat terrain, the Bi-LSTM model showed better daily streamflow prediction performance in such regions. As forest cover increased, the predictive performance of the hybrid model gradually declined. Moreover, the hybrid model performed better in test areas with higher imperviousness, indicating that the model had better predictive accuracy for highly urbanized watersheds. When the test area's forest cover exceeded 20%, the model's performance rating started to decline. Similarly, when the imperviousness exceeded 30%, the model showed more accurate predictions of daily streamflow. Among the 14 test areas, the highest accuracy prediction had an NSE of 0.92 and a PBIAS of 1.34%, corresponding to regions with forest cover ranging from 2 to 5% and imperviousness ranging from 47 to 54%. This indicates that the hybrid model meets the demand for daily streamflow prediction in this area, showing high predictive accuracy with no significant systematic bias, and can provide a good hydraulic environment for subsequent simulations of BMPs' pollution reduction effects.

**Figure 7:**
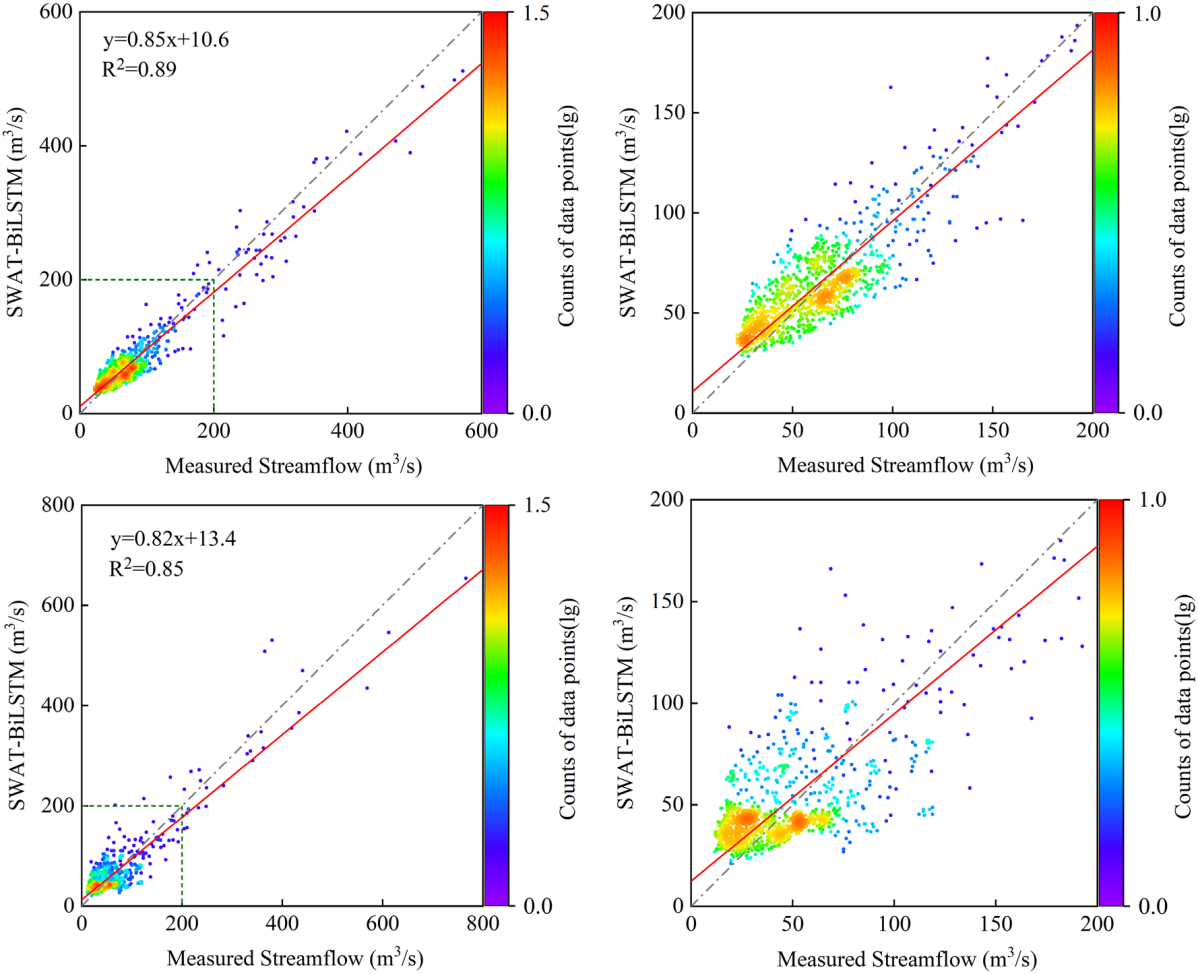
Scatterplots between measured and simulated daily streamflow at the outlet of BRB. The top two figures show the calibration period and the bottom two figures show the validation period.

Figure 7 shows the simulation performance of the hybrid model for total streamflow in the BRB during both the calibration and validation periods. The figure also displays the fitted linear regression line and R^2^ between simulated and observed data. Throughout the simulation process, the hybrid model exhibited an underestimation trend for daily streamflow exceeding 200m^3^/s. For daily streamflow below 200m^3^/s, the performance of the hybrid model during the validation period was relatively worse compared to the calibration period, with the flow data points scattered more widely around the 1:1 line. As a data-driven model, Bi-LSTM's performance is greatly influenced by the input data used to train the model. If the training data is not representative, the performance of Bi-LSTM may not meet expectations. In this study, only daily streamflow data from the calibration period were used to train the Bi-LSTM model, which may be a reason for the hybrid model's poorer performance during the validation period. Nevertheless, considering the overall distribution of daily streamflow data points, they are evenly scattered on both sides of the 1:1 line without showing any significant systematic bias trend, which still meets the requirements for establishing a water environment model. Accurate streamflow simulation results contribute to better estimates of pollutant loads and ensure the precise modeling of pollutant transport processes, which are essential input data for water quality models. In this study, the SWAT-BiLSTM model's ensemble average of performance metrics for the water quality simulation results in the BRB is presented in Table 9. During the calibration and validation periods, the model achieved NSE and R^2^ values exceeding 0.8 for TN and TP simulation results. This indicates a high level of consistency between the simulated results and the actual values. Additionally, the absolute values of PBIAS were all below 7%, suggesting that the model did not exhibit significant systematic bias in simulating water quality.

**Table 9:** Summary of calibration and validation statistics for TP and TN.

Type	Calibration period (2015–2019)			Validation period (2020–2022)		
	*NSE*	*R*^2^	*PBIAS* (%)	*NSE*	*R*^2^	*PBIAS* (%)
TN	0.88	0.83	−4.71	0.84	0.80	−6.58
TP	0.81	0.80	−2.47	0.82	0.82	−5.92

### Efficiencies of individual BMP scenarios in reducing NPS pollution loads

**Figure 8:**
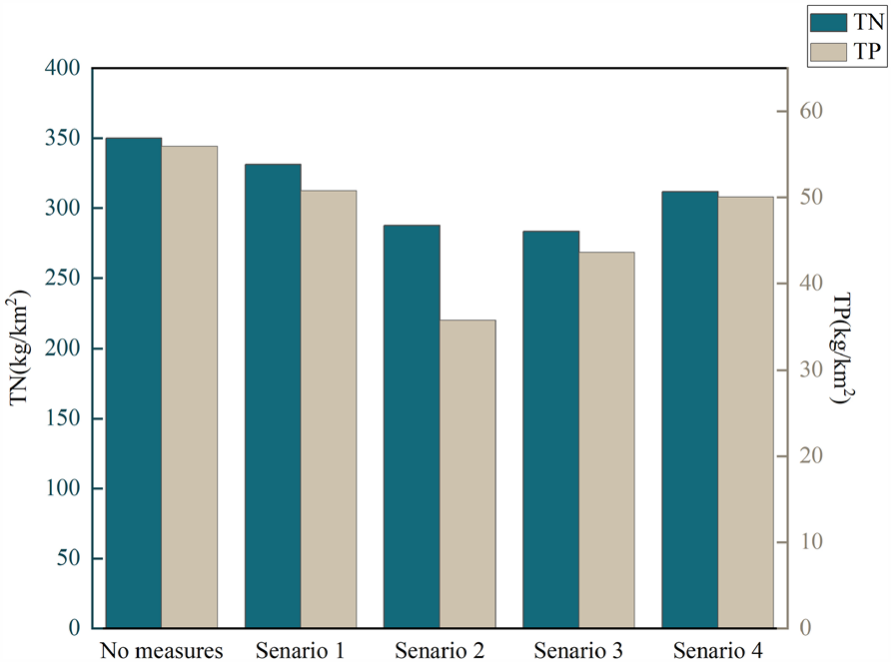
Effectiveness of overall pollution load reduction in the BRB under four single-measure scenarios.

Figure 8 illustrates the reduction effects of BMPs on TN and TP under four scenario with single BMP measure. In scenario 1, the average reduction rates of TN and TP pollutant loads across the entire watershed by adopting Formula Fertilization by Soil Testing were 5.36% and 9.18%, respectively. Overall, this measure showed a better reduction effect on TP than on TN. The main reason for this result is that the major land use type in the BRB is rainfed agriculture, which is more prone to phosphorus runoff. The results indicate that Formula Fertilization by Soil Testing can reasonably reduce the use of chemical fertilizers with minimal impact on crop yields while reducing the pollution load. However, the overall reduction effect on TN and TP is limited, with average reduction rates within 10% throughout the watershed. In scenario 2, stubble mulch resulted in an average reduction rate of 17.83% for TN and 36.17% for TP across the entire watershed, indicating a significant reduction effect on nitrogen and phosphorus losses. Moreover, this measure showed a better reduction effect on TP than on TN, which may be attributed to the dominant soluble phosphorus pollution in the study area, which is carried into water bodies through surface runoff. Previous research suggested that stubble mulch can reduce about 60% of surface runoff, preventing pollutants from entering water bodies with surface runoff. Therefore, this measure exhibited a better reduction effect on TP in the study area. In scenario 3 and scenario 4, VFS and Grassed Waterways demonstrated average reduction efficiencies of 19.07% and 10.95% for TN, and 22.02% and 10.52% for TP, respectively, across the entire watershed. The results indicate that Vegetative Buffer Strips were more effective than Grassed Waterways, possibly due to their significant sediment interception effect, reducing the entry of particulate pollutants attached to sediment into water bodies. Among all single BMP scenarios, stubble mulch showed the best reduction effect on TN and TP in the BRB and is considered one of the BMP strategies that farmers can easily implement. It can be prioritized when planning to adopt a single BMP measure for controlling NPS pollution emission in the BRB.

### Efficiencies of combined BMP scenarios in reducing NPS pollution loads

**Figure 9:**
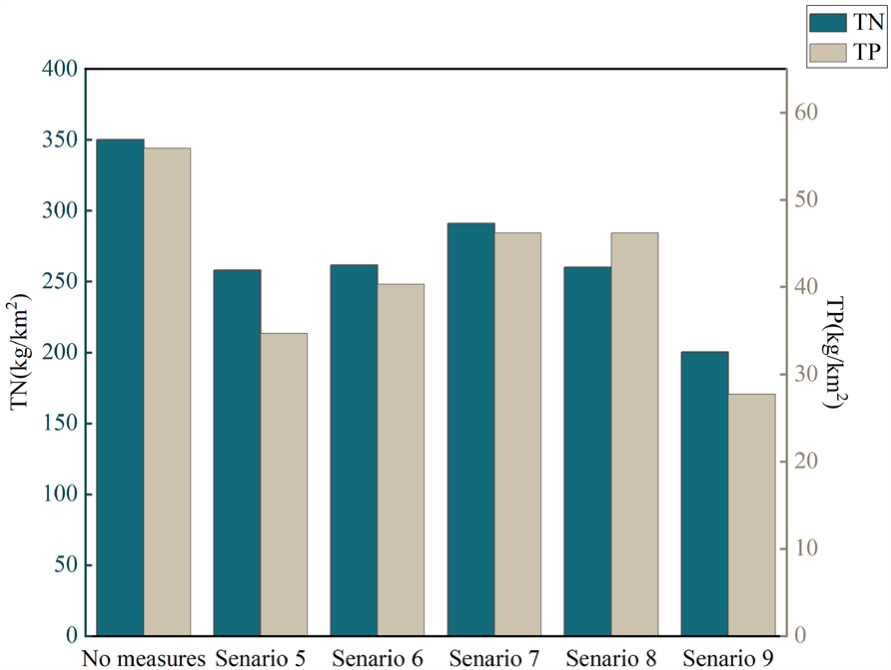
Effectiveness of overall pollution load reduction in the BRB under five combined-measure scenarios.

Considering that optimization design often involves implementing multiple BMPs to achieve the reduction goals for multiple pollutants, BMP combination scenarios should be designed to assess the overall effectiveness of various BMPs. The reduction rates of TN and TP under combined BMP scenarios are shown in Fig. 9. Among the five combination scenarios, the combination of Formula Fertilization by Soil Testing, stubble mulch, and VFS demonstrated the best reduction effect, achieving reduction rates of 42.71% for TN and 50.40% for TP. Other combination scenarios also showed favorable reduction effects. Overall, the combined BMPs scenarios exhibited higher average reduction rates for TN and TP compared to single BMP scenarios, with an increase of 13.75% and 15.27%, respectively. The combination of agricultural BMPs and structural BMPs proved to be more effective in controlling NPS pollution in the BRB.

## Conclusion

In this study, a highly functional framework combining SWAT and Bi-LSTM models was developed to explore the effectiveness of different BMP scenarios in reducing NPS pollution in areas without measured runoff data. In this approach, SWAT served as a transfer function to convert meteorological data into baseflow and stormflow, which were then used as inputs for the Bi-LSTM model. The model performance was evaluated using three metrics: NSE, R^2^, and PBIAS. The results showed that the hybrid model achieved an NSE and R^2^ of 0.88 and 0.89, respectively, during the calibration period, and both remained above 0.85 during the validation period. The absolute maximum PBIAS was 2.71%, indicating that the hybrid model has high predictive accuracy without significant systematic bias, meeting the demand for simulating NPS pollution emission control schemes. The partial calibration of SWAT model parameters and coupling with the Bi-LSTM model helped address the uncertainty caused by equifinality in the SWAT calibration process. This framework provides a promising approach for simulating NPS pollution emission control schemes in other regions without measured streamflow data.

Based on the hybrid model, the hydrodynamic environment established, and the control effect of different BMP scenarios on NPS pollution in the BRB evaluated. The results showed that stubble mulch and vegetative filter strips were more effective in reducing pollutants than formula fertilization by soil testing and grassed waterways, reducing TN loads by 17.83% and 19.07%, and TP loads by 36.17% and 22.02%, respectively. Stubble mulch demonstrated the best overall reduction effect for both TN and TP, being farmer-friendly and prioritized for single BMP-based NPS pollution control plans. Furthermore, compared to single BMP scenarios, combined BMP scenarios increased the average reduction rates of TN and TP by 13.75% and 15.27%, respectively. The combination of VFS, formula fertilization by soil testing, and stubble mulch showed the best reduction effect, with reduction rates of 42.71% for TN and 50.40% for TP. These results provide powerful support and evidence for decision-makers in formulating NPS pollution emission control schemes for the BRB.

The hybrid model combining SWAT and Bi-LSTM simplified the hydrological processes and made some assumptions, introducing uncertainty to predictions. In the future, more advanced deep learning models or hybrid models could be explored, combining various modeling methods to better simulate complex hydrological processes and achieve more accurate predictions in areas without measured streamflow data. Additionally, more types of BMPs and pollutants can be considered in further research to promote practical applications. The coupling of models such as vine copulas could also be used to predict the probability of achieving emission control goals with various combined BMP scenarios.

## Data Availability

The datasets used and/or analysed during the current study available from the corresponding author on reasonable request.
